# NMR Profiling of Reaction and Transport in Thin Layers: A Review

**DOI:** 10.3390/polym14040798

**Published:** 2022-02-18

**Authors:** Ruben Nicasy, Henk Huinink, Bart Erich, Adan Olaf

**Affiliations:** 1 Applied Physics Department, Eindhoven University of Technology, P.O. Box 513, 5600 MB Eindhoven, The Netherlands; r.j.k.nicasy@tue.nl (R.N.); s.j.f.erich@tue.nl (B.E.); o.c.g.adan@tue.nl (A.O.); 2 Organization of Applied Scientific Research, TNO The Netherlands, P.O. Box 49, 2600 AA Delft, The Netherlands

**Keywords:** high-resolution nuclear magnetic resonance (NMR), thin layer, transport, reaction, GARField, STRAFI, MOUSE

## Abstract

Reaction and transport processes in thin layers of between 10 and 1000 µm are important factors in determining their performance, stability and degradation. In this review, we discuss the potential of high-gradient Nuclear Magnetic Resonance (NMR) as a tool to study both reactions and transport in these layers spatially and temporally resolved. As the NMR resolution depends on gradient strength, the high spatial resolution required in submillimeter layers can only be achieved with specially designed high-gradient setups. Three different high-gradient setups exist: STRAFI (STRay FIeld), GARField (Gradient-At-Right-angles-to-Field) and MOUSE (MObile Universal Surface Explorer). The aim of this review is to provide a detailed overview of the three techniques and their ability to visualize reactions and transport processes using physical observable properties such as hydrogen density, diffusion, $T_{1}$- and $T_{2}$-relaxation. Finally, different examples from literature will be presented to illustrate the wide variety of applications that can be studied and the corresponding value of the techniques.

## 1. Introduction

Thin layers (10–1000 µm) are very important in a broad range of applications [1] that can be found in building materials [2], energy storage [3,4], photovoltaic devices [5,6], art [7,8], flexible electronics [9], optics [10] and most importantly in coatings [11,12,13]. The reason to incorporate thin layers can be manifold, for example, down-scaling [14], introducing novel technologies [15], increasing performance and stability. Another important feature when using thin polymer films is the ability to incorporate a huge variety of functional molecules which allow electrical [16,17,18], magnetic [19] or even color-like [20] like features in the thin layer. Performance and stability of these thin layers will be determined by their final structure and underlying physical processes. In literature, the study of these processes is mostly categorized in different groups belonging to polymer or non-polymer thin layers. Here, we also differentiate between polymer and non-polymer films. Within the polymer films, we identify two main groups of processes, firstly, the ones during film formation and, secondly, processes that occur when the film is formed. The process of film formation is a multistage process which in literature can be defined in different ways. In this review, we categorize two types of film formation, both starting with the evaporation of the solvent followed by either curing or coalescence. After the film has formed, other processes such as transport of liquid and chemical reaction take place. In non-polymer films, such as cements or printing paper, liquid uptake and deformation are the most important processes. A more fundamental understanding of these processes will lead to more cost-effective, efficient and stable applications.

Film formation [21,22] is crucial in determining the final structure of the film and its stability. If film formation happens incorrectly, the layer can suffer from non-uniformities, skin formation [23,24], precipitation [25], chemical differences and other mechanical malfunctions [26] that all reduce performance. In most cases, applying thin layers starts with a liquid solution that covers the surface, containing both the film material (polymer, latex) and a solvent [21]. In the mid-1900s, volatile organic components were mostly used as solvent. However, since 1950, concerns about their toxicity [27], flammability and environmental effects [28,29] have led to a change towards using water as solvent [30]. When the layer is applied, film formation happens in a two-stage process where first the solvent has to evaporate, allowing close contact between the polymers, followed by a type of mechanism to overcome their mutual repulsion and form a rigid layer. The rate of solvent evaporation is critical for film formation and depends on multiple factors [31,32]. At first, the rate of evaporation will depend on vapor pressure, temperature, surface area and air flow. In a second stage, the release of solvent becomes limited by transport through the thin layer [31,33]. If the solvent evaporates too quickly, the polymer will not have time to adhere to the surface or form a continuous film. In contrast, a slow evaporation rate will induce overwetting in the subsurface. When enough solvent has evaporated, a continuous layer is formed by curing (cross-linking) or coalescence in the case of latex [34,35]. Both cross-links and coalescence will be responsible for the strength and characteristics of the initial thin layers. A wide variety of studies focus on cross-linking [36,37,38], and coalescence [22] to gain a better understanding and improve the final structure.

Processes after film formation such as liquid transport [39,40] and reactions within these films are responsible for performance, degradation and instabilities. A wide variety of degradation processes can be identified, including biodegradation [41], photodegradation by light [42] and chemical degradation. A more fundamental understanding of the transport of liquids and chemical reactions within the thin layer will contribute to better performance and stability of the thin layer. Studies on the penetration and diffusion of liquids and the corresponding effect on the film morphology are the focus of a large group of studies and will be discussed in this review [43].

Other types of non-polymer thin layers can be found in the printing industry [44,45,46] (printing paper with a thickness of 100 µm). In these printing papers, the penetration of ink is of great importance as it influences the final print quality [47,48]. The wide variety of coatings [49], hydrophobicity [50] and basis weight found in these papers will all influence the printing process and the resulting print quality. Another type of thin layer can be found in the human skin [51,52] where layers such as the stratum corneum and viable epidermis, both around 50 µm thick, can be found. In these skin layers, processes such as the penetration of skin care products [53] or oils take place. As a final example, in cement pastes [54,55] used in building applications, strength is based upon the internal morphology and transport of ions.

Experimental techniques able to study film formation, reaction and transport processes are necessary to gain the insights allowing improvement of performance and stability. Since many processes in thin layers such as diffusion, curing and chemical reactions will happen with a so-called front, gaining spatial and time-dependent information is a crucial requirement. Conventional techniques are not able to gain both spatial and time-resolved information coherently. Techniques such as weight measurements [56], microscopy or ASA (Automatic Scanning Absorptometer) measurements [57] can only measure global properties such as mass or liquid uptake in the case of ASA, but will not gain spatial information. High-spatial-resolution MRI has proven to be a versatile tool for studying these processes. Nuclear Magnetic Resonance (NMR) is a well-established technique that started in 1946 where for the first time NMR was used for the detection of magnetic properties of atoms by Bloch [58,59] and Purcell [60]. The method was later extended to two different areas, namely, nuclear Magnetic Resonance Imaging (MRI) and NMR spectroscopy, which led to Nobel Prizes in Medicine [61] (Paul C. Lauterbur and Sir Peter Mansfield) and Chemistry [62] (Richard R. Ernst and Kurt Wuthrich). In this review, the focus lies on the first one (MRI).

MRI has made its way into material research, as well as in polymer research [22,63,64,65,66]. NMR can be used to measure density profiles, atoms’ mobility, and probe their environment which enables to study reactions and transport processes in thin layers. Reactions in thin films, such as cross-linking [67,68,69], glass transitions, curing and many more chemical reactions, will all influence the mobility of the atoms, which is visible in the NMR signal. Additionally, transport processes can be studied by measuring time-dependent density profiles. We will discuss different studies to demonstrate how chemical and physical information of thin polymer films between 10 and 1000 µm can be extracted using high-spatial-resolution NMR setups.

Characterizing polymers with NMR has been the focus of many studies. Most research is conducted with NMR spectroscopy which is already well known and described in many books [70,71,72,73]. Here, the focus lies upon the different high-resolution NMR setups that are able to extract information with high spatial resolution. Three different high-gradient NMR setups exist with resolution in the micrometer range that are able to extract physical and chemical information about thin films: STRAFI (STRay FIeld), GARField (Gradient-At-Right-angles to Field) and MOUSE (MObile Universal Surface Explorer). This paper will begin by explaining the basic theory behind NMR and how hydrogen atoms can be used to extract spacial information.

In Section 3, the GARField [74], STRAFI [75] and MOUSE [76] setup will be discussed, where an overview will be given about their similarities and differences. Lastly, in Section 4, different thin layer studies will be discussed in order to provide a better understanding of the methods’ possibilities.

## 2. Theory

### 2.1. NMR Spatial Encoding

The existence of a nuclear spin was demonstrated by Bloch and Purcell in 1946 when they measured for the first time nuclear magnetic resonance (NMR) [58,77]. They received the Nobel Prize in Physics for this work in 1952. The basics of NMR are often explained using a classical vector model [65]. In this classical model, the nuclei can be described by a small magnetic dipole with magnetic moment $\vec{\mu }$ [Am${}^{2}$], also known as “spin”. The nuclear spin $\vec{\mu }$ is the consequence of a moving charge within the atoms nucleus. A relation exists between the angular momentum $\vec{b}$ and the magnetic moment of the nucleus $\vec{\mu }$,

(1) $$\vec{\mu }=\gamma \vec{b}$$

where the proportionality constant γ [rad s${}^{-1}$ T${}^{-1}$] is the gyromagnetic ratio. The most studied nucleus (also abundant in polymers) is hydrogen (${}^{1}$H) where γ/2π = 42.58 MHz/T. We want to emphasize that although hydrogen is the most abundant and well known atom, there also exist studies on other elements such as fluorine [78] (40.08 MHz/T) or carbon-13 (10.71 MHz/T) [79].

When these nuclear spins enter an external applied magnetic field $\vec{B_{0}}$ [T], they will experience a torque $\vec{\tau }$ [Nm] related to the magnetic field by

(2) $$\vec{\tau }=\vec{\mu }\times \vec{B_{0}}$$

As the torque equals the time derivative of the angular momentum $\vec{b}$ Equations (1) and (2) can be combined to:

(3) $$\frac{d\vec{\mu }}{dt}=\gamma \vec{\mu }\times \vec{B_{0}}$$

**Figure 1:**
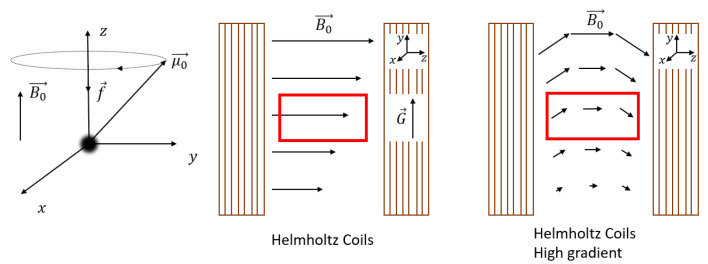
**Left**: Larmor frequency, middle: conventional NMR setup (Helmholtz coils) with condition |B|/G≫1 where the gradient field $\vec{G}$ and applied magnetic field $\vec{B_{0}}$ are shown and approximated by straight lines because the radii of curvature in this case are negligible. Depicted in red is the measurement area used in NMR experiments. **Right**: The same Helmholtz coils as the middle, but with |B|/G≪1. In this particular case, the radius of curvature cannot be neglected which can be seen in the field lines from $\vec{B_{0}}$. $\vec{G}$ is not drawn because it lies in correspondence with the middle figure, not in one direction, which would make the graph too complicated.

Since all the nuclei will experience this same time dependence in their magnetic moment $\vec{\mu }$, they will start to align and precess around the applied magnetic field $\vec{B_{0}}$, which by normal convention is pointing in the *z*-direction. This precession is called Larmor precession. A schematic picture of this precession is shown in Figure 1 left. The frequency $\vec{f}$ [MHz] depends on the magnetic field and is given by

(4) $$\vec{f}=\frac{\vec{\omega }}{2\pi }=\frac{\gamma }{2\pi }\vec{B_{0}}$$

where $\vec{\omega }$ [rad s${}^{-1}$] is the Larmor frequency.

By applying a second magnetic field $\vec{B_{1}}$ perpendicular to the main magnetic field ($\vec{B_{0}}$), the hydrogen atoms will precess along the new established magnetic field which allows manipulation of the hydrogen atoms. The excitation is best when the radio-frequency (RF) pulse exactly coincides with the Larmor frequency $\vec{\omega }$ from Equation (4). In most cases, this manipulation is used to bring the magnetic moments of the hydrogen atoms in the xy-plane. In an NMR measurement, the total magnetisation $\vec{M}$ along this xy-plane can be measured, which is a sum of all the magnetic moments,

(5) $$\vec{M}=\sum_{i}\vec{\mu_{i}}$$

Measuring this signal is mostly conducted in the form of spin echoes, firstly introduced by Hahn [80,81] in 1950. After a combination of pulses that will be discusses in Section 2.3, the signal is recorded at some echo time $t_{e}$[s].

One of the advantages of NMR is the ability to achieve spatial resolution along the *y*-direction, perpendicular to the thin layer. Encoding spatial information can be achieved by applying a magnetic field gradient $\vec{G}\left[T/m\right]\equiv \left(\partial B_{z}/\partial x,\partial B_{z}/\partial y,\partial B_{z}/\partial z\right)$ perpendicular to the $\vec{B_{0}}$ field, see Figure 1 middle. Depending on the strength of the gradient (*G*) and magnetic field ($|B_{0}|$), there exist two different scenarios, |B|/G≫1 and |B|/G≪1.

The rest of this section will cover the special case where |B|/G≫1, found in conventional NMR setups. In this particular case, the curvature of the magnetic field lines is so small that the following assumptions can be made: the magnetic field is constant, pointing in the *z*-direction, and the gradient along the *y*-direction can be assumed to be constant and equal to $\partial B_{z}/\partial y$ (Figure 1 middle). Both assumptions will simplify the explanation. However, for most high-gradient NMR setups used for thin film profiling where |B|/G≪1 [74], these assumptions are not valid anymore. This will be discussed in Section 2.4.

In the case where |B|/G≫1, the gradient will cause a change in magnetization along the *y*-direction, indicated by the black arrows in Figure 1 middle, which leads to a change in Larmor frequency,

(6) $$\omega \left(y\right)=2\pi f\left(y\right)=\gamma \left(B_{0}+G_{y}\cdot y\right)$$

with $G_{y}$—the gradient of the *z*-component of the magnetic field along the *y*-direction ($\partial B_{z}/\partial y\approx \partial B_{0}/\partial y$) and *f*—the frequency amplitude. The total measured NMR signal is a combination of all different nuclei that resonate with different Larmor frequencies. When applying a Fourier transform of the recorded echo, the separate contributions of all different Larmor frequencies can be extracted and linked to a specific *y*-position through Equation (6), providing the spatial density distribution ρ(y). After excitation, the total signal intensity $S(y,t_{e})$ at position *y* will decay over a time due to environmental effects and is given by

(7) $$S\left(y,t_{e}\right)\propto \rho \left(y\right)\left(1-\exp\left(\frac{-t_{r}}{T_{1}}\right)\right)\left(\exp\left(-\frac{t_{e}}{T_{2}^{*}}\right)\right)$$

where $T_{1}$ and $T_{2}^{*}$ are characteristic timescales that define the signal decay. More information about these times and the underlying processes can be found in the next section.

### 2.2. NMR Relaxation

In the previous section, the formula for the signal intensity (Equation (7)) introduced two characteristic time scales that described signal loss after excitation. This signal loss is known as relaxation and will be described in this section. There are two typical characteristic relaxation processes called $T_{1}$- or spin–lattice relaxation and $T_{2}$- or spin–spin relaxation.

In the first one, energy between the hydrogen spins and the surroundings is exchanged. This energy exchange will relax the spins back to their original *z*-direction. How fast $M_{z}$ is restored is characterized by this $T_{1}$-time. The energy transfer will be most effective when surrounding atoms vibrate at the Larmor frequency. The second relaxation process will account for losses in the transversal component of the magnetization vector ($M_{xy}$). The rate at which this relaxation occurs is defined by a $T_{2}^{*}$-relaxation time that is strongly correlated to the molecular motion and the local environment. For example, dipolar interactions between neighboring atoms induces a small difference in magnetic field [82], which results in dephasing and a faster signal decay.

Both relaxation times will be influenced by different local factors that enable to extract physical information about their surroundings and their mobility. To understand how these times can be used to gain physical information, a deeper look at both relaxation times is provided.

The total $T_{2}^{*}$- and $T_{1}$-relaxation time can be written as the contribution of different factors as [83]

(8) $$\frac{1}{T_{2}^{*}}=\frac{1}{T_{2}}+\frac{1}{T_{2i}}=\frac{1}{T_{2dip}}+\frac{1}{T_{2pores}}+\frac{1}{T_{2dif}}+\frac{1}{T_{2i}}$$

(9) $$\frac{1}{T_{1}}=\frac{1}{T_{1dip}}+\frac{1}{T_{1pores}}$$

where $T_{2}^{*}$ and $T_{1}$ are the relaxation times found in Equation (7). Here, $T_{2}^{*}$ is a combination of both $T_{2}$ and $T_{2i}$, where the second one is attributed to inhomogeneities in the magnetic field introduced by the setup [84]. When using Hahn [80,81], CPMG [85,86] or other pulse sequences (Section 2.3), one can compensate for field inhomogeneities and the $T_{2i}$ contribution can be neglected. Therefore, $T_{2}^{*}$ can be replaced by $T_{2}$.

**Figure 2:**
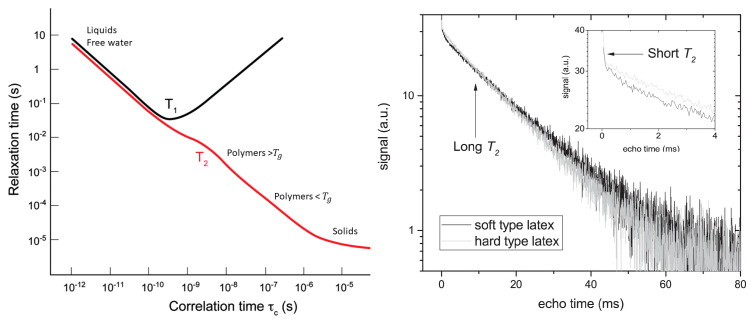
**Left**: Dependence of $T_{1}$ and $T_{2}$ upon $\tau_{c}$ (Reproduced from Chem. Soc. Rev., 2014,43, 1627–1659 with permission from the Royal Society of Chemistry.) [106]. **Right**: Signal decay of a latex film measured with a GARField NMR. Both soft and hard-type latex are visible and show a multi-exponential decay where the short and long $T_{2}$– component are marked. The short relaxation time is attributed to hydrogen atoms within the polymer, while the long relaxation time comes from free water. (Reprinted from *Progress in Organic Coatings*, Volume 123, Benjamin Voogt, Henk Huinink, Bart Erich, Jurgen Scheerder, Paul Venema and Olaf Adan, Water mobility during drying of hard and soft type latex: Systematic GARField ${}^{1}$H NMR relaxometry studies, Pages 111–119, Copyrights 2018, with permission from Elsevier [107]).

The first relaxation mechanism comes from neighboring dipoles ($T_{1dip}$, $T_{2dip}$). Dipoles will introduce small changes in the local magnetic field that lead to a faster relaxation. In most cases, this relaxation mechanism can be linked to the molecular mobility. This molecular mobility is quantified by a motional correlation time ($\tau_{c}$) which is defined as the average time necessary for a molecule to rotate one radian. The influence of dipole–dipole interaction was described by Bloembergen, Purcell and Pound (BPP) in 1948 [87], where they established relationships between $T_{1dip}$ and $T_{2dip}$ and the motional correlation time ($\tau_{c}$), Figure 2 left:

(10) $$\frac{1}{T_{1dip}}=\frac{3}{10}\frac{\gamma^{4}\hbar^{2}}{r^{6}}\left[\frac{\tau_{c}}{1+\omega_{0}^{2}\tau_{c}^{2}}+\frac{\tau_{c}}{1+4\omega_{0}^{2}\tau_{c}^{2}}\right]$$

(11) $$\frac{1}{T_{2dip}}=\frac{3}{20}\frac{\gamma^{4}\hbar^{2}}{r^{6}}\left[3\tau_{c}+\frac{5\tau_{c}}{1+\omega_{0}^{2}\tau_{c}^{2}}+\frac{2\tau_{c}}{1+4\omega_{0}^{2}\tau_{c}^{2}}\right]$$

These relaxation times can be used to discriminate between different polymer motional regimes inside a thin film, providing information about different structures, mobility, chemical reactions and mechanical responses. In a rigid environment, such as solids or polymers below their glass transition temperature, hydrogen atoms will experience the same deviations in magnetic field for a longer time, leading to a larger phase difference and faster decay. On the contrary, when molecules are mobile, for example in liquid water, the differences in magnetization will be averaged out over time leading to a longer relaxation time. This was used to observe for example different polymer substances [66], crosslinked and non-crosslinked materials and skin layers within thin polymer films [88,89], see also Section 4. This could ultimately be used to observe any difference between materials where the molecular mobility of the studied atoms is different.

The second term appearing in both relaxations is the relaxation caused by a porous matrix ($T_{1pores},T_{2pores}$) which was described in detail by Brownstein and Tarr [90]. When atoms diffuse through a porous matrix they will encounter the pore surface, where relaxation due to dipoles or other effects occurs. How effectively this surface relaxation occurs depends on the particular medium and is quantified by the surface relaxivity σ [m/s] [91,92,93,94,95]. Depending on the diffusion constant (*D* [m${}^{2}$/s]), pore radius (*r*[m]) and surface relaxation, different regimes can be excluded [96]. There is a fast diffusion regime with σr/D≪1, where the surface relaxation will become dominant, and a slow diffusion regime, with σr/D≫1 where the surface relaxation can be neglected. Here, the factor r/D is related to the number of encounters with the surface. Therefore, the surface relaxation will become important when the surface relaxivity is high or many encounters are present. In the fast diffusion regime, Brownstein and Tarr found that $1/T_{1pores}$ and $1/T_{2pores}$ can be approximated by

(12) $$\frac{1}{T_{2pores}}=\sigma_{2}\frac{S}{V}$$

(13) $$\frac{1}{T_{1pores}}=\sigma_{1}\frac{S}{V}$$

where the factor S/V is the pore surface-to-volume ratio. These relaxation times can be used to gain useful information about the porous matrix [83,91,92,93,94,97] and possible changes over time when subjected to different environmental factors.

A third parameter only found in $T_{2}$-relaxation comes from diffusion of molecules. When atoms diffuse, they will encounter a different field strength due to the setups gradient. This leads to dephasing and an accelerated decay of $M_{xy}$. This diffusion-induced decay will also influence the resulting $T_{2}$-time. This effect will become larger with increased gradient strength, diffusion constant (*D*[m/s${}^{2}$]) and diffusion time ($t_{e}$), which is described by the following formula [98,99],

(14) $$\frac{1}{T_{2}dif}=\alpha \gamma^{2}G^{2}t_{e}^{2}D$$

Probing relaxation times can also be useful for studying different pools of atoms at the same time, a trick commonly used in thin film studies [100,101,102,103,104]. When the relaxation times of different hydrogen groups are far enough apart, their relative contributions to the signal can be separated. Figure 2 right demonstrates this principle by showing a decay of a latex film [105]. The decay clearly shows a two-component exponential decay. From the BPP-theory we know that the latex can be attributed to short relaxation time while the long $T_{2}$-relaxation time can be linked to free water.

By exploiting this method, the relative contribution and relaxation time of different pools of hydrogen atoms can be followed over time using the same experiment. This is by far the most powerful tool in studying thin films over time [108]. Using this technique, M.R.Halse [109] showed that they could follow three groups of hydrogen atoms in a decane/rubber system at the same time: dry rubber ($T_{2}=0.7$ ms), swollen ($T_{2}=1.8$ ms) rubber and solvent ($T_{2}=200$ ms).

### 2.3. Pulse Sequences

In the beginning, the magnetization vector is in equilibrium, pointing in the same direction as the main magnetic field $\vec{B_{0}}$. Bringing the magnetisation out of equilibrium and creating an NMR signal is achieved by using a radio frequency (RF) wave. This RF irradiation will have a magnetic component ($\vec{B_{1}}$) along the xy-plane exerting a torque on the magnetization (Equation (2)). Applying this RF-field for a certain amount of time is called an “RF-pulse”. The angle by which the magnetization will rotate is called the “flip angle” (α[${}^{\circ }$]) and depends on both the pulse time ($t_{p}$[s]) and the magnitude of the RF-field ($B_{1}$[T]) given by

(15) $$\alpha =\gamma B_{1}t_{p}$$

Applying different RF-pulses is referred to as a pulse sequence and can be used to measure all the above mentioned parameters such as signal intensity, relaxation times and diffusion. As the focus of this review lies on the techniques, only the main sequences and some basics will be discussed. More details and explanations on the different types of pulse sequences can be found in more advanced studies [43,88,110] and reviews [83,111].

Measurements of the signal intensity are mostly performed using Hahn spin echoes [80,81] with the following pulse sequences [90${}^{\circ }$-τ-180${}^{\circ }$-τ-echo] [111]. At *t* = 0, a 90${}^{\circ }$ pulse rotates the spins into the xy-plane. After this pulse, the transverse magnetization starts to dephase due to the environment, field inhomogeneity and the gradient according to Equation (7). At *t* = $t_{e}/2$, a second 180${}^{\circ }$ is given that refocuses the spins. At *t* = $t_{e}$, the spins are refocused and a so-called spin echo is created from which the transverse magnetization can be measured.

To measure the $T_{2}$-relaxation, the Carr–Purcell–Meiboom–Gilll sequence (CPMG) [85,86] is performed. The sequence start exactly the same as the Hahn spin echo, followed by a train of 180${}^{\circ }$ pulses [90${}^{\circ }$-τ-(180${}^{\circ }$-τ-echo-τ)${}_{n}$]. Every $180^{\circ }$ pulse refocuses the spins, resulting in a series of spin echoes. The echo intensity drops due to $T_{2}$-relaxation. The signal intensity at the *n*th echo is given by

(16) $$S\left(nt_{e}\right)=S_{0}\exp\left(-nt_{e}/T_{2}\right)$$

from which the $T_{2}$-relaxation time can be calculated. In literature, adjustments to this well-known sequence are reported. A specific pulse sequence that should be mentioned here is the Ostroff–Waugh (OW) pulse sequence where the 180${}^{\circ }$ pulse is exchanged by another 90${}^{\circ }$ pulse [112]. In high-gradient measurements, this sequence is chosen above the more conventional CPMG sequence, for reasons that will be explained in Section 2.5.

To measure the $T_{1}$-relaxation time, conventional NMR setups use mainly two sequences, the saturation recovery and inversion recovery sequence. However, in the high-resolution setups described in this review, the saturation recovery sequence is chosen for reasons that will be explained in Section 2.5. The pulse sequence of the saturation recovery sequence is given by [(α)${}_{m}$-$\tau_{2}$-90${}^{\circ }$-τ-180${}^{\circ }$-τ-echo] [113]. The measurement starts by saturating the nuclear spins, setting the magnetization in the transverse plane to zero. This is achieved by *m* pulses with a certain angle α. After complete saturation, Hahn spin echoes will be measured at different time intervals specified by $\tau_{2}$. The measured echo intensity is then given by

(17) $$S\left(\tau_{2}\right)=S_{0}\left(1-\exp\left(-\tau_{2}/T_{1}\right)\right)$$

from which the $T_{1}$-relaxation time can be calculated.

### 2.4. High Resolution Spatial Encoding

To profile thin films with a high-enough resolution, a high gradient is required, see Equation (6). Three NMR setups are able to generate high-enough gradients to profile films between 10–1000 µm, namely, STRAFI (STRAy FIeld) [114], GARField (Gradient-At-Right-angles-to-Field) [74] and MOUSE (MObile Universal Surface Explorer) [115]. In these high-gradient setups, where |B|/G≪1, Equation (6) cannot be used anymore because the curvature in the magnetic field lines is significant, see Figure 1 right.

To understand this, we need to consider Maxwell in free space with a static magnetic field and no current,

(18) $$\nabla \times \vec{B}=0$$

According to this condition, the following relation should hold for the gradient in the *y*-direction ($G_{y}$ = $\partial B_{z}/\partial y$),

(19) $$\frac{\partial B_{z}}{\partial y}=\frac{\partial B_{y}}{\partial z}$$

**Figure 3:**
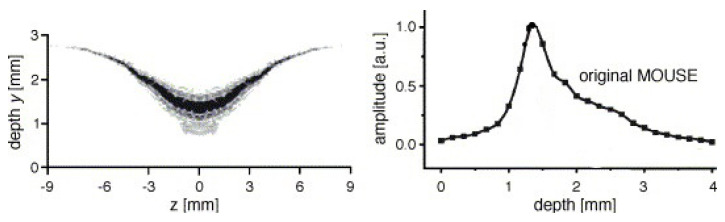
**Left**: sensitive region of an original NMR-MOUSE. **Right**: Slice thickness of the signal amplitude for a conventional NMR-MOUSE (Reprinted from *Magnetic Resonance Imaging*, Volume 23, Issue 2, Bernhard Blümich, Federico Casanova, Juan Perlo, Sophia Anferova, Vladimir Anferov, Kai Kremer, Nicolae Goga, Klaus Kupferschläger, Michael Adams, Advances of unilateral mobile NMR in nondestructive materials testing, pages 197–201, Copyright 2005, with permission from Elsevier. [116]).

Therefore, by introducing a gradient in the *y*-direction ($\partial B_{z}/\partial y$), there must be a gradient in another direction ($\partial B_{y}/\partial z$) resulting in a curvature into the mean magnetic field, which is of the order of |B|/G [74]. In conventional NMR setups with a low gradient, this curvature is around $10^{2}m$ and can be neglected as in the previous section. When the gradient becomes much larger, the assumptions made in the previous section are not valid anymore. Consequently, it follows from Equation (6) that

(20) $$\vec{f}\left(\vec{r}\right)=\frac{\gamma }{2\pi }\left(\vec{B_{0}}+\vec{G}\cdot \vec{r}\right)$$

where we use a vector notation to account for the fact that the Larmor frequency is different along the same horizontal plane. As signals are excited and resolved according to their respective Larmor frequency, this inhomogeneous Larmor frequency will lead to some problematic effects within high-resolution setups. The RF-pulses that normally excite a rectangular region will now excite curved slices that depend on the shape of the magnetic field lines. The curvature in STRAFI, for example, is around 0.1 m [74], much lower than in conventional NMR setups (10${}^{2}$ m). Figure 3 shows the sensitive region of an original NMR-MOUSE where a clear curvature can be observed [116].

### 2.5. Resolution and Field of View

In the previous section, we discussed how a gradient is used to encode spatial information in a measurement. The resulting resolution is mostly determined by the high gradient and its ability to encode and read out spatial information. However, there are some other limiting factors that determine the maximum achievable resolution of the experimental setup. Another important parameter is field of view (FOV) which provides the maximum area that can be measured with the NMR setup. This section will cover some of the most important factors that determine the final resolution and FOV. Both parameters will be discussed for two measurement strategies—a Fourier measurement and a slice selective pulse measurement. In the first one, the spatial information is collected via a Fourier transformation of the NMR signal, mostly found in GARField [105,117] and sometimes in STRAFI [118]. In the slice selective pulse measurement, a slice selective pulse will collect the NMR signal of a thin slice without the need for a Fourier transformation. Here, the profile is built up slice-by-slice which requires a mechanical movement of the sample or magnet, mostly found in STRAFI [119] and MOUSE [120].

In a Fourier transformation measurement, the maximum theoretical resolutions that can be achieved are determined by the lowest frequency difference (Δf [Hz]) that can be differentiated. This difference will depend on the window width or acquisition time ($\Delta t_{a}$ [s]) of the measurement ($\Delta f\approx 1/\Delta t_{a}$) [121]. The dimension that corresponds to this frequency difference is determined by the gradient [121] and is given by

(21) $$\Delta z=\frac{1}{\gamma G_{z}\Delta t_{a}}$$

Thus, for a gradient of 40 T/m and a window width of 100 µs, the maximum achievable resolution becomes 5.9 µm. It can therefore be seen that both a higher gradient or acquisition time will increase the maximum theoretical resolution. However, $\Delta t_{a}$ should always be lower than the $T_{2}^{*}$ found in Equation (8). When $\Delta t_{a}>T_{2}^{*}$, the signal is limited by a fast $T_{2}$-relaxation and not by the acquisition time. In this particular case, $\Delta t_{a}$ in Equation (21) should be replaced by $T_{2}^{*}$. As the typical $\Delta t_{a}$ of high-gradient setups are around 100 µs, much lower then conventional NMR setups, these cases almost never appear.

In the slice selective measurements, the resolution is determined by the frequency bandwidth (Δf) of the RF-pulse. The frequency bandwidth from the RF-pulse is inversely proportional to the pulse length ($t_{p}$). Thus, for a 10 µs pulse time, the excited frequency bandwidth (Δf) is around 0.1 MHz which for a gradient of 40 T/m would excite a slice of 58.7 µm = ($\Delta r\approx 1/t_{p}\gamma G$).

A problem arises in high-gradient fields because of the curvature induced on the magnetic field (see Section 2.1) that limits the homogeneity of $|\vec{B_{0}}|$ and therefore the resolution. In the STRAFI and MOUSE, these inhomogeneities will be the limiting factor for the resolutions. Determining these resolutions can be achieved experimentally by measuring the profile of a thin slice, containing NMR active atoms, and analyzing the resulted profile [3]. An example of this limitation is shown in Figure 3, where on the left the sensitive area for a normal NMR-MOUSE is shown. The corresponding depth profile can be viewed on the right. In most cases, the experimental resolution is determined by taking the half-width of this profile which in this case would be around 1 mm and limits the maximum achievable resolution. As will be explained later, GARField introduces specially designed poles in order to make $|\vec{B_{0}}|$ homogeneous [74]. For this reason, the achievable resolution in GARField is not limited by inhomogeneities and is mostly higher than in a STRAFI and MOUSE setup.

The high gradients will also induce a large spread in resonant frequencies and unlike a conventional NMR imaging setup, the gradients cannot be switched off. The spread in resonance frequencies can be of the order of 25 MHz cm${}^{-1}$ [121] which severely limits the single-shot field of view (FOV) that can be achieved with one RF-pulse. In a Fourier measurement, the theoretical FOV is determined by the slice selective pulse ($\Delta r\approx 1/t_{p}\gamma G$) which can be increased by lowering the pulse time. Now, we can explain why in a high-gradient setup, an OW-sequence and saturation recovery sequence are chosen above the more conventional CPMG sequence and inversion recovery sequence. Since the OW- and saturation recovery sequences use only 90${}^{\circ }$ pulses, always the same volume will be excited. In a CPMG- and inversion-recovery sequence where 90${}^{\circ }$ and 180${}^{\circ }$ pulses are used, this is not the case. Another problem in the case of the inversion recovery arises in the first pulse, used to invert the magnetization from $M_{z}$ to $M_{-z}$, which will not be exactly 180${}^{\circ }$ throughout the sample which can interfere with the $T_{1}$-measurement. However, in real measurements, limitations arise because the sensitivity drops significantly when moving away from the RF-coil, limiting the FOV for a GARField setup to around 500 µm depending on the signal-to-noise ratio.

In a slice selective measurement, the sample can be moved through the sensitive area which in essence could result in a limitless FOV. However, the setup design will mostly be the limiting factor leading to a FOV in the order of a few millimeters.

The slight differences between the three setups will lead to slight changes into the achievable resolution and FOV. A more detailed explanation can be found in Section 4 where the setups are discusses separately. However, in most cases, the achievable resolution and FOV can be approximated using the above-mentioned formulas.

## 3. Methods

In this section, the three mentioned high-resolution NMR setups (STRAFI, GARField and MOUSE) will be discussed in more detail. The goal is to provide a general guideline that helps in deciding which setup is most suitable for studying a specific application or material.

### 3.1. STRAFI (STRAy Field Imaging)

**Figure 4:**
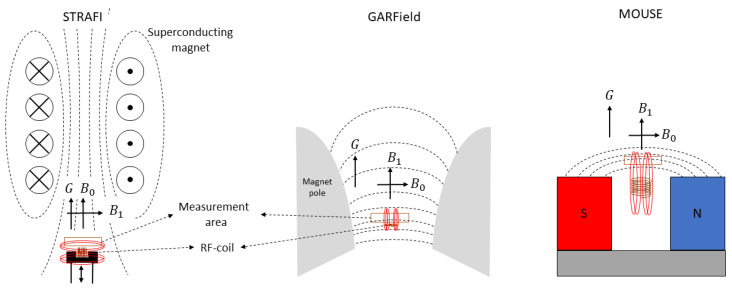
**Left**: schematic representation of STRAFI. Middle: schematic representation of GARField. **Right**: schematic representation of MOUSE. In all figures, the measurement area is depicted with an orange rectangle. The magnetic field lines are indicated with black for the $B_{0}$-field while the ones from the RF-pulse are indicated with red. In all setups, the direction of the gradient (*G*), main magnetic field ($B_{0}$) and RF-pulse field ($B_{1}$) in the measurement area are indicated with black arrows.

Stray field imaging was introduced by A. A. Samoilenko et al. [75] in 1988. The STRAFI technique uses the stray field (fringe field) of a superconducting magnet to produce its high gradient. Figure 4 left is a schematic representation of a STRAFI setup. Indicated with black dotted lines are the field lines from the superconducting magnet indicating the direction of the main magnetic field $\vec{B_{0}}$. The measurement area lies in the stray field just below the superconducting magnet where a large gradient can be found that lies along the same direction as $\vec{B_{0}}$. Typical gradients that can be achieved are between 30 and 60 [Tm${}^{-1}$] [114]. Indicated with red lines in Figure 4 are the field lines from the RF-pulse. From here there exist mainly two different ways to acquire a complete profile, leading to two types of STRAFI, conventional STRAFI [122,123] and Fourier transform STRAFI [118].

In the conventional STRAFI, the profile is acquired by recording the NMR signal slice-by-slice [122,123]. One method of imaging slice-by-slice is by moving the sample, which can be seen in Figure 4. As slices are imaged separately, no Fourier transform is required, which leads to a resolution that is limited by the frequency bandwidth of the excitation pulse, see Section 2.5. The fact that the resolution is pulse-time-dependent involves some drawbacks and limitation on the resolutions. As the pulse lengths can never be longer than the $T_{2}$ of the material, the resolution for polymers with very short $T_{2}$ times is limited. Since the frequency bandwidth of the pulse is inversely proportional to the pulse time, this method allows to have rather good resolutions at the expense of long measurement times. Another time restraint comes from the mechanical movement required in slice-by-slice measurements that has a huge influence on the measurement time. This becomes problematic when measuring fast dynamic processes. However, there are some tricks to lower the time required to measure a single profile. For example, during the repetition time, one can already start to measure other slices which means that the time is limited by the $T_{1}$ of the sample [114]. As slices are measured separately and changing, some parameters will always influence measurement time. For example, increasing the resolution or sample thickness will also increase measurement time as more slices need to be measured. Therefore, for slow processes, good resolution can be achieved, while for fast processes, the measurement time is too long. Thus, measuring with STRAFI is easier for slow processes and lower resolutions (above 50 µm).

In the special case, when the frequency band with of the excitation pulse is large enough to capture the complete sample, a Fourier transform STRAFI can be implemented [118]. Here, the echo is collected from the whole sample and the profile is reconstructed by a Fourier transform. Using this method, measurements are only limited by $T_{1}$ and not because of mechanical movement. This technique was mostly used to image thin film below 400 µm. The theoretical resolutions in these measurements are determined by Equation (21). However, the resolution is mostly limited by the shape of the sensitive area and should be determined experimentally. A major drawback of STRAFI is that the sensitive area where the $\vec{B_{0}}$-field is most homogeneous does not coincide with the optimum gradient position. Therefore, the actual gradient is mostly lower than the maximum gradient that is reported for the particular electromagnet used in the STRAFI setups. After a trade-off between measurement speed and resolution, the most encountered studies reported resolutions between 50 [124]–450 [122] µm.

### 3.2. GARField (Gradient-At-Right-Angles-to-Field)

To solve the high-curvature problem found in STRAFI that limits the maximum resolution, the GARField NMR was introduced in 1999 by P. M. Glover et al. [74]. In this setup, the researchers changed from a gradient along one component (*y*-direction) to a gradient in the magnetic field amplitude $|\vec{B_{0}}|$. To achieve this, the setup uses electromagnets with specially designed pole tips. The specific shape was calculated by P.M. Glover et al., using a specific solution of the Laplace equation $\Delta^{2}\phi =0$ where ϕ is the scalar potential defined by B= grad ϕ. For a detailed calculation, we refer to the original paper [74]. The specially designed pole tips from the electromagnets are able to generate a horizontal plane where $|B_{0}|$ is constant along the xy-plane and perpendicular to the gradient, Figure 4 middle. When making these pole tips, it was found that the ratio G/|B| is always constant and independent of *y* and *z*. This allows to operate with different gradient strengths without losing the in-plane uniformity of the magnetic field, allowing for an easy adaptation of the resolution. Shown in Figure 4, middle, is a schematic representation of the setup where the shape of the magnetic pole tips can be seen. Implementing these magnetic poles was only possible when the orientation of the magnetic field and gradient were slightly different compared to STRAFI. Where in STRAFI the $B_{0}$ and *G* are aligned, they are perpendicular in the GARField setup. As the RF-pulse should be perpendicular to the main magnetic field, the RF-coil from the STRAFI and GARField will have different orientations. The highest sensitivity is obtained when the sample is placed at the end of the RF-coil. This is only possible in the GARField setup. In STRAFI, this arrangement is impossible and the sample should be placed apart from the RF-coil, which lowers the sensitivity.

Compared to conventional STRAFI and MOUSE, a depth profile is measured in one single measurement without repositioning the sample or magnet. The time required to measure a single profile depends on the different parameters used in the pulse sequence but is typically shorter than in STRAFI. A drawback, however, is the limited FOV. Without the possibility to reposition the sample, the FOV is mostly limited by the reduced sensitivity when moving away from the coil, mostly around 500 µm.

As some applications such as coatings are mostly used on metallic surfaces, a special note should be made. Metallic surfaces interfere with the magnetic signals in the setup, which introduces artifacts in the measurement. Artifacts can be the result of differences in magnetic susceptibility between polymer and metal and from eddy currents generated in the metal. To address this problem, H. Zhu et al. [125] investigated the effect of metal substrates on the NMR signal. The researchers found through simulations and experimental work that magnetic susceptibility can be neglected when measuring with a GARField NMR, but that the eddy currents interfere with the pulse field. Therefore, it was calculated that measuring on metallic surfaces required more pulse power to manage these eddy currents.

### 3.3. The MOUSE

Different from the STRAFI and GARField, the NMR-MOUSE (MObile Universal Surface Explorer) is a portable device invented to investigate large objects in a nondestructive manner [7,8]. The NMR-MOUSE consists of a compact permanent magnet (red and blue in Figure 4), which generates the $B_{0}$-field. Inside the magnets, the magnetic field is homogeneous but when moving to the fringe field, the field starts to become more inhomogeneous, creating a rather high gradient of about 22 T/m depending on the specifics of the magnets [7]. The small size and low weight make it suitable for on-site testing in a non-destructive manner. This makes the NMR-MOUSE suitable to carry out measurements on large surfaces such as walls or paintings without the need for collecting samples and destroying the object.

This magnetic field is typically quadratic along the *x*- and *z*-direction and with a main gradient along the *y*-direction. This variation in the *y*-direction can be used to extract depth profiles of a specific material. The $B_{1}$-field is obtained from a built-in RF-coil, see Figure 4 right. By calculating the exact magnetic field, is has been found that there is a sensitive region just outside the device [126]. Just like a conventional STRAFI, slice-selective pulses are used to extract spatial information, therefore, recording a signal is fully equivalent to STRAFI [114]. However, the sensitive region lies outside the magnets, which limits the final depth that can be measured. Different devices are available to measure different depths ranging from 3 to 25 mm [7]. Selecting the best device depends on the application as more depth coincides with a lower sensitivity.

A drawback of the simple magnetic design is the fact that the sensitive region is oddly shaped, varying in thickness, see Figure 3. This results in low sensitivity and huge restrictions on the maximum achievable resolution. Spatial resolutions better than half a millimeter are hard to achieve [127,128]. Attempts have been made in order to reduce the resolution. J. Perlo et al. [129] reported resolutions as low as 2.3 μm by making a new magnetic geometry with four permanent magnets. Measuring with these high resolutions in a slice-selective manner is, however, very time consuming. Another drawback of the design is the limitation of the sensitive volume, which limited the FOV to 50 µm.

### 3.4. Guideline

Choosing the proper setup depends on multiple parameters, such as the sample material, required resolutions and measurement time.

When resolution is most important, GARFIeld is the best option. By solving the curvature problems found in STRAFI, GARField will have the best resolution of all setups—the most reported resolutions lie somewhere between 5 and 15 µm (Table 1). In addition to a good resolution, the Fourier measurements used in GARField offers faster measurement speed then slice-selective measurements (Table 1). Profiles are mostly measured at times between 1 and 10 min. It should be mentioned that STRAFI also has a Fourier implementation, but with lower resolutions (most reported resolutions between 24–60 µm) and lower sensitivity coming from the RF-coils orientation. Therefore, for a dynamical process or if a high resolution is required, GARField would be the best option. A drawback of GARField is the rather low FOV (<500 µm) with a sensitivity that lowers when moving away from the RF-coil.

When larger samples should be measured, STRAFI would be a better choice. Due to the slice-selective excitation, samples can be moved through the sensitive area, making the FOV, in essence, limitless. While at 400 µm the GARField already loses a lot of sensitivity, the STRAFI does not encounter this signal loss. In these measurements, resolution always comes with a trade-off towards measurement time. Higher resolutions require to measure more slices when keeping the FOV constant.

When measurements are required on-site or if a small piece of the sample is not available, MOUSE is the only suitable option. For most applications such as walls, oil/water wells [130,131,132], PE pipes [116] or paintings, samples small enough for the STRAFI or GARField setup are not available, leaving MOUSE as the only option.

An overview of the most frequently encountered parameters is given in Table 1. It should be mentioned that in all cases, trade-offs between different parameters are made. Therefore, the best achievable resolution is almost never achieved. However, the table should give an idea about different ranges that are commonly used for the different setups.

## 4. Applications

The following sections will provide a deeper look into the most common types of measurement performed by these three NMR setups on thin layers. Every section will start by discussing the general concepts using a representative study as an example, followed by a summary of related studies on a wide variety of samples and materials.

### 4.1. Structure and Structural Evolution

Studying thin layers starts by identifying their internal structure and structural evolution. Therefore, the first NMR measurements performed on thin layers aimed to determine the moisture content and different polymer states such as crystallinity or cross-linking. Determining the structure of thin layers can be achieved using the signal intensity, diffusivity and $T_{1}$/$T_{2}$-relaxation times. All these parameters provide information about the hydrogen content, their environment and micro structures, as described in Section 2.2. In the first part of this section, two studies on cement pastes performed by P. J. McDonald et al. [54,55] are used to explain how the relaxation times can be used to obtain structural information, followed by a summary of some important studies using similar principles. In these studies, GARField was only used to measure in one particular slice. In principle, this information could have been obtained via conventional NMR instruments. Nevertheless, we value this particular study as GARField has the potential to perform a similar study, but with high spatial resolution in depth. Lastly, a study performed by B. Voogt et al. [107] is used to explain how diffusivity can be used to determine structural information using the above mentioned setups.

**Figure 5:**
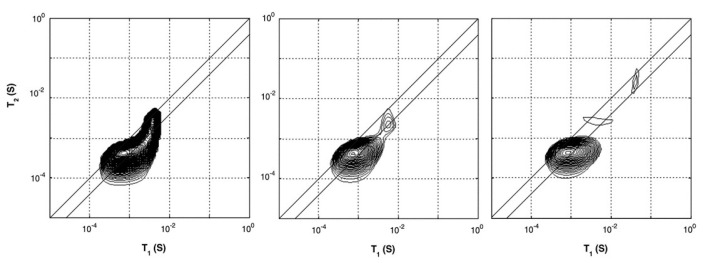
$T_{1}$-$T_{2}$-correlation maps of white cement with water–cement ratio of 0.4. Figures are taken from the curing process under water after 2 days (**left**), 6 days (**middle**) and 7 days (**right**). A development in pore space can be observed by the separation of the single peak into smaller peaks when approaching 6 and 7 days. (Reprinted from *Magnetic Resonance Imaging*, Volume 25, Issue 4, Peter J. McDonald, Jonathan Mitchell, Michael Mulheron, Luc Monteilhet, Jean-Pierre Korb, Two-dimensional correlation relaxation studies of cement, pages 470–473, Copyrights 2007, with permission from Elsevier [54]).

In studies performed by P. J. McDonald et al. [54,55], the researchers characterized hydrated cement paste using different $T_{1}$- and $T_{2}$-relaxation studies. $T_{1}$- and $T_{2}$-relaxation times for different cement paste were determined using a saturation recovery and OW-sequence with the GARField-setup. Using the GARField-setup, a slice selective measurement was performed 10 mm below the surface of the sample with a slice thickness of 0.6 mm. When plotted in a $T_{1}$-$T_{2}$ correlation spectrum, the researches could relate the relaxation times to different groups of hydrogen atoms with varying pore radii (Equations (12) and (13)). Such plots for a white cement sample cured under water are shown in Figure 5. They identified that the cement paste had pores with the following length scales: gel pores ($T_{2}$ = 400 µs) and multiple capillary pores ($T_{2}$ > 400 µs) with different pore sizes. Observing the different relaxation times, they found that after 6 days, 2 peaks are visible, both with low $T_{1}$ and $T_{2}$ times corresponding to gel pores. At day 7, capillary pores with longer relaxation times also start to become visible (Figure 5). Characterizing bulk properties like this can also be performed using a normal NMR spectrometer [133]. In this particular case, the researchers identified the correlation maps at a specific location of 10 mm below the surface. The advantage of the GARField NMR could be to perform the correlation maps at different positions and study the different pore fraction at different positions, however, this was not done in this particular study.

**Figure 6:**
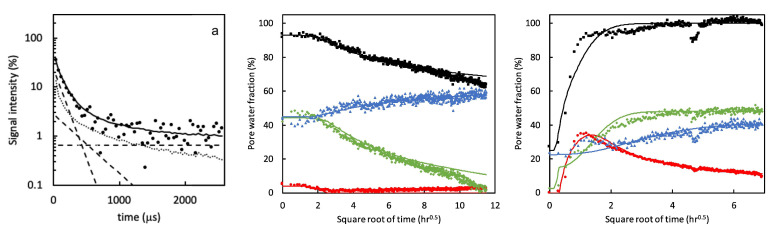
**Left**: Signal intensity decay measured by a quadrature echo train. The solid line is the total fit. The dashed lines represent the fractions with different $T_{2}$ times (120 µs, 360 µs and 1080 µs). Middle and right: Pore water fractions measured 600 µm below the surface during drying (**middle**) and wetting (**right**). Red corresponds to water in capillary pores, green to gel sized pores and blue to interlayer spaces. Black represents the total hydrogen content. (Reprinted from *Cement and Concrete Research*, Volume 133, Peter J. McDonald, Ors Istok, Magdalena Janota, Agata M. Gajewicz-Jaromin, David A. Faux, Sorption, anomalous water transport and dynamic porosity in cement paste: A spatially localised ${}^{1}$H NMR relaxation study and a proposed mechanism [55]).

In a follow-up study [55], the dynamic porosity in cement paste during water uptake and drying was studied, using $T_{2}$-relaxation. Three main groups were found that correspond to different pore sizes: hydrates inner layers of 1 nm ($T_{2}$ = 120 µs), gel pores of 3–5 nm ($T_{2}$ = 360 µs) and capillary pores larger than 5 nm ($T_{2}$ = 1080 µs). Figure 6, left, shows typical signal decays where the solid line is the total decay and marked with dashed lines are the contributions of the different pores. For completeness, the authors added a dotted line representing the part coming from the crystalline solid. Using this multi-exponential decay, the researchers could follow the different types of hydrogen atoms during evaporation (Figure 6 middle) and moisture uptake (Figure 6 right). Red corresponds to water in capillary-sized pores, green to gel-sized pores and blue for interlayer pores and black is the total amount of moisture. The authors observed that after 1 hour, the moisture uptake already reaches 90% of its total amount. A striking fact is that they saw for the first time that moisture will start to redistribute between the different pores. In this research, they only looked at a particular part in the layer, however, with GARField, MOUSE or STRAFI, the same information at different positions is available at every position in the layer.

Studies on cement like-materials such as pastes and Portland cement have also been performed on STRAFI [134,135,136] and MOUSE [135,137]. In other studies, this separation based on relaxation times was used to separately study water and polymer contribution in latex films [105,107], human skin [138] and another group of polymers such as Polyurethane coatings [139], plasticizing of nylon-6 [140,141], semi-crystalline polyethylene [142] and photo-polymerization of methacrylate [143]. Additionally, medical applications, such as the binding behavior of collagen-binding liposomes, have been studied [144,145]. Even layers with very low relaxation times, such as ice ($T_{2}$ = 3.5 µs), have been imaged during melting using a STRAFI setup [122].

**Figure 7:**
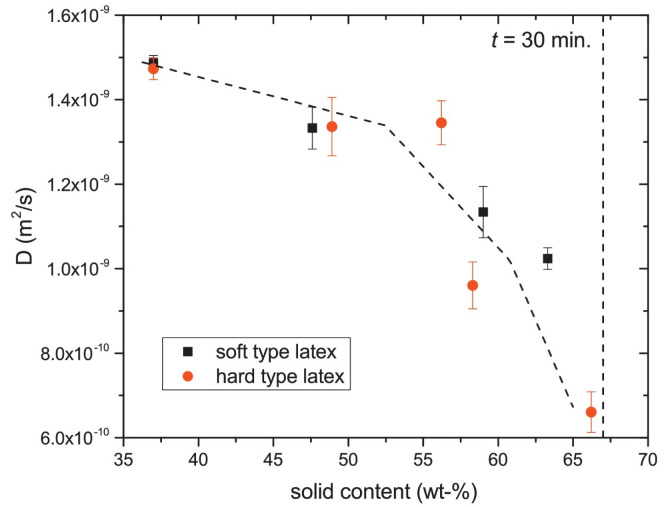
Diffusion coefficient of free water within the hard- and soft-type latex. (Reprinted from *Progress in Organic Coatings*, Volume 123, Benjamin Voogt, Henk Huinink, Bart Erich, Jurgen Scheerder, Paul Venema and OlafAdan, Water mobility during drying of hard and soft type latex: Systematic GARField ${}^{1}$H NMR relaxometry studies, Pages 111–119, Copyrights 2018, with permission from Elsevier [107]).

In addition to $T_{2}$- and $T_{1}$-relaxation, another way to characterize the structure of thin layers is via the diffusion coefficient *D* of the hydrogen atoms. In materials, water can be present in a wide variety of states with different diffusion coefficients such as free water, water confined in pores or water bound to the polymer matrix. When materials undergo a structural transition, the water diffusivity will be altered, giving insight into the internal structure of the layer. A study performed by B. Voogt et al. [107] demonstrates this principle. The researchers used the proton local mobility and diffusivity to characterize the structure of hard and soft latex during drying. By measuring the $T_{2}$-relaxation time with different echo times, the diffusion coefficient was determined, see Equation (14). Figure 7 shows the diffusion coefficient measured at different times steps during drying. From *t* = 30 min onward, the diffusion constant was too low to be determined. As a result of drying, the particles’ mobility will be restricted lowering the auto-diffusion *D* (Figure 7) and the $T_{2}$ of the atoms. The $T_{2}$- of latex remained rather constant around 0.1 ms, indicating that the proton mobility is constant. By analyzing the diffusion constants and relaxation times, they found that water changes from a free state towards pore water and finally water that is physically bound to the polymer matrix. The researchers also observed a difference in packing between the soft and hard type of latices. The relative concentration of the polymer increased in the soft type but remained constant in the hard type. This indicates that in the soft type, the particles come closer together, forming a close packed structure, while for the hard type, this is not possible due to the hardness. These measurements clearly show the restricted movement of the hydrogen atoms.

In a similar study performed by V. Baukh et al. [141], multilayered coatings were investigated. The authors found that the diffusion coefficient increased with water content and that binding of water to the polymer was stronger at low water concentrations. In other studies, diffusion coefficients were measured in order to determine the amount of bacteria [123] or structural changes in water-swollen cellophane [146].

As the diffusion coefficient depends on the structure of the material, it can also be used to differentiate layers in heterogeneous structures. P. J. McDonald et al. [52] used this method to determine the diffusion constants and profiles of the human skin in vivo. The measurements revealed a clear contrast between the stratum corneum and viable epidermis. This contrast was largely attributed to a difference in diffusion constant between both layers. Comparable studies on human skin that characterized the different skin layers were performed on GARField [51,147] and MOUSE [138,148]. In addition to water and polymers, studies on small penetrants such as toluene and n-hexane in PE pellets have also been performed [149].

### 4.2. Film Formation—Drying

Film formation is the process in which a polymer emulsion or colloidal dispersion will overcome their stabilizing forces to form a continuous film. These stabilizing forces can be overcome when the continuous liquid phase evaporates, forcing the emulsion droplets or polymer particles together. The evaporation of excess liquid is referred to as the drying phase of the film formation process. When enough liquid has evaporated, the polymers or colloidal particles need to form a continuous film. After solvent evaporation, there are two types of processes: curing and coalescence [150]. In the curing process, a stable film is formed by cross-links between reactive polymers. On the other hand, in coalescence, the polymer particles will first coalescence and deform to form a close packed structure. In order to form a homogeneous film, the deformation is followed by entanglement of the polymer chains. Particle boundaries disappear, which will lower the film roughness [22]. All these combination of drying, curing and coalescence make film formation a complicated process. Since most applied films and coatings undergo this film formation, this section is dedicated to studies that are focused this topic. First, drying experiments and then the subsequent processes are discussed.

**Figure 8:**
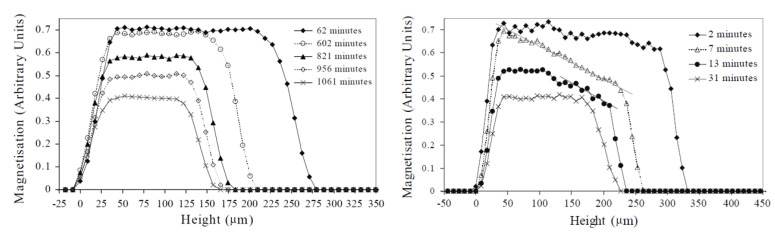
NMR profiles measured during film drying of an alkyd emulsion in a closed environment with Pe = 0.2 (**left**) and with Pe = 16 (**right**). Additionally, the water fractions where determined for every profile, where for the left figure the water fraction were 0.48 (62 min), 0.33 (602 min), 0.23 (821 min), 0.15 (956 min) and 0.09 (1061 mi.), while for the right figure, the water fractions were 0.44 (2 min), 0.25 (7 min), 0.15 (13 min) and 0.10 (31 min). (Reprinted by permission from Springer Nature Customer Service Centre GmbH Springer Nature: *The European Physical Journal* E—Soft Matter, Vertical water distribution during the drying of polymer films cast from aqueous emulsions, J.-P. Gorce et al., 2014 [151]).

During drying, water or solvent evaporates from the polymer film, mostly reducing the overall thickness. The drying process can be monitored with an NMR signal. A representative example of a study on the drying processes of waterborne colloidal films was performed by J.-P. Gorce et al. [151]. Measurements were performed on a GARField setup using an OW-sequence with a 8.7 μm resolution. Profiles of the NMR signal measured at different times are shown in Figure 8. The width of the signal corresponds to the thickness of the polymer film. First of all, evaporation leads to film shrinkage due to the disappearance of hydrogen atoms. When all water has evaporated, the profile reaches its final thickness, corresponding to 150 µm. This decrease in profile thickness can be used to characterize a drying front and a corresponding drying rate. Secondly, the maximum signal intensity reduces over time from 0.7 to 0.4. To understand the reduced signal, we need to introduce ρ = $\rho_{a}+\rho_{b}$ in Equation (7),

(22) $$S\propto \rho_{a}\exp\left(-t_{e}/T_{2a}\right)+\rho_{b}\exp\left(-t_{e}/T_{2b}\right)$$

where we have left out the $T_{1}$-term. Here, the signal is split into a term coming from the solvent ($\rho_{a}$, $T_{2a}$) and one coming from the polymer or colloidal particles ($\rho_{b}$, $T_{2b}$). When solvent is released, the total active hydrogen atoms ρ is almost unchanged as hydrogen atoms from the water are replaced by the ones from the polymer. Therefore, the signal decrease cannot be attributed to a loss in active hydrogen atoms. However, the relative contributions do change, where during drying $\rho_{a}$ reaches zero, $\rho_{b}$ reaches its maximum. As the mobility of the polymer is much lower then that of free water, the $T_{2}$ of the polymer is lower then that of free water by at most one order of magnitude (see Section 2.2). Faster relaxation leads to a lower signal, where the final signal amplitude is closely related to the mobility of the polymer phase which in this particular case was very low. In the beginning, the signal intensity (0.7) is dominated by the solvent ($\rho_{a}\exp\left(-t_{e}/T_{2a}\right)$); after drying, the signal will be determined by the polymer ($\rho_{b}\exp\left(-t_{e}/T_{2b}\right)$) which due to a faster decay will have a lower signal intensity (0.4).

In the same study, the influence of the Peclet number Pe = $vL_{ef}/D$ was investigated. v[m/s] is the speed of the receding water front, $L_{ef}\left[m\right]$—the thickness of the layer and $D[m^{2}/s]$—the diffusion coefficient. By controlling the thickness, the speed of the receding water front and the diffusion coefficient, the Pe number could be adjusted. When Pe > 1, advection dominates, but for Pe < 1, diffusion is more important. Figure 8 left shows profiles for the case were Pe = 0.2, whereas right represents Pe = 16. A difference in profile shape was observed that indicates that diffusion towards the surface can keep up with the evaporation (flat profiles) when Pe = 0.2, but not when Pe = 16, resulting in a concentration gradient of water molecules near the surface. The formation of this dense polymer layer to the surface showed limited diffusion towards the surface, slowing down evaporation.

In a comparable study performed by P. Ekanayake et al. [152], density profiles of drying colloidal films revealed that the particle concentration gradient inside this dense layer of colloidal particles scales with $Pe^{0.8}$. Furthermore, in this study, the GARField setup was chosen as it has the best resolution, allowing to see this thin dense top layer.

In some cases, drying can induce the formation of a skin layer that can trap water. These skin layers reduce solvent evaporation and significantly slow down the drying process [23,24]. E. Ciampi et al. [23] investigated skin formation upon drying of poly(vinyl alcohol) (PVOH). Profiles were measured with a GARField NMR for an initial PVOH content of 10 WT% and 25 WT%. The profiles for the 10 WT% dried similarly to the ones measured by [151]. In case of high-polymer-weight fractions (25 WT%), a skin layer developed that trapped water inside the profile, leading to slower evaporation.

Similar studies of the drying behavior of different polymers, such as alkyd layers [89,117,153,154], latex layers [107], gelatin layers [155] and even dental resins [119,156,157,158], have been conducted using the GARField, STRAFI and MOUSE NMR setups.

### 4.3. Film Formation—Curing

**Figure 9:**
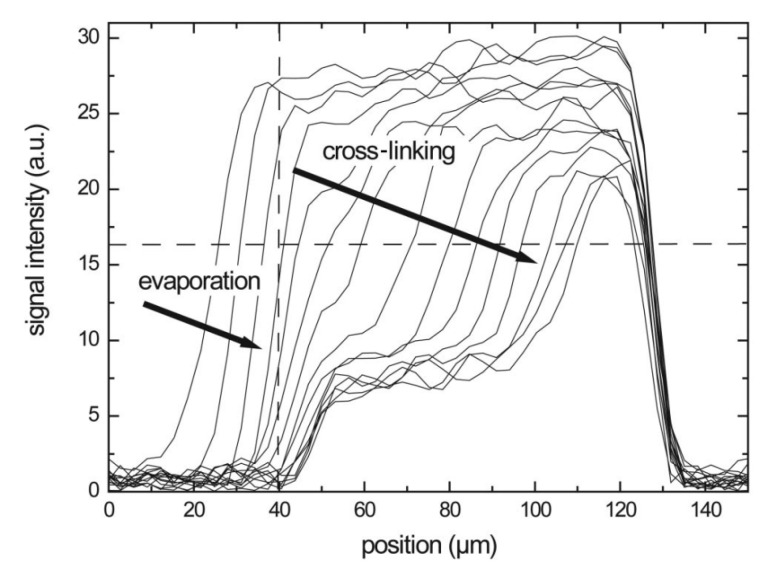
Hydrogen profiles of a solvent-borne alkyd sample measured during drying. The top of the coating corresponds to the left side, while the cover glass can be found on the right. The vertical dashed line indicates the top of the film after drying. (Reprinted from S. J. F. Erich, J. Laven, L. Pel, H. P. Huinink, and K. Kopinga, “Dynamics of cross linking fronts in alkyd coatings”, *Appl. Phys. Lett.* 86, 134105 (2005) https://doi.org/10.1063/1.1886913, with permission of AIP Publising [153]).

In case of reactive compounds, a curing reaction can start when enough water evaporates and the polymer content is high enough. Measurements with a GARField on drying alkyd coatings [153] illustrate the capability to measure the drying–curing film formation process. In this study performed be S. J. F. Erich et al., NMR profiles on alkyd coatings were measured every 10 min with an OW-sequence. Measured profiles can be seen in Figure 9. The profiles reveal this two-stage process where the polymer layer shrinks and the intensity drops due to solvent evaporation, as explained in previous section. This process is indicated by the arrows in Figure 9 and it accounts for a rapid loss in signal intensity in the beginning. After drying, a (reaction) curing front moves into the polymer film. The signal loss due to curing of polymers can be attributed to the loss in mobility of the polymer. When polymers are cured, they become more rigid, the $T_{2}$ drops and signal is lost when the $T_{2}$ approaches $t_{e}$.

In the study of S. J. F. Erich et al. [153], curing experiments are performed on water-borne and solvent-borne alkyds. Both systems showed the same behavior: a clear evaporation and curing stage could be distinguished. In a related paper [89], NMR and confocal Raman Microscopy were compared, and the curing could be related to the disappearance of double bonds and the formation of oxidative cross-links. These cross-links lowered the mobility and reduced the NMR signal, giving rise to the observed curing front. Tracking these fronts in different environmental conditions revealed that oxygen supply was the limiting factor in the curing front dynamics.

Further, the effect of drying on porous media [117], catalysts [159,160,161,162] and pigment volume concentrations [163] was studied.

Curing also plays a role in dental resins. By exposing the resin to a light source, it will begin to polymerize and shrink. This polymerization shrinkage (PS) is crucial for the durability of the material, as it will determine stresses and strains in the film and the uptake of fluids and bacteria. A polymer that is widely used as a dental bonding agent is dimethacrylic acid. Curing studies on this polymer have been performed with a STRAFI NMR [119,156,157,158]. In an experiment performed by T. Nunes et al. [119], glass vials were filled with a liquid resin. The liquid resin was exposed to light. Different groups of hydrogen atoms could be discriminated based on their $T_{2}$- and $T_{1}$-relaxation times: mobile molecules (free monomers) with long relaxation times and rigid molecules (cured AB2). It was found that oxygen strongly influenced the kinetic behavior. Using STRAFI, the influence of different cements and curing protocols could be determined and all were shown to have an effect on the particular curing process, and they should be chosen with care in medical applications.

Other curing measurements can be found for the curing of wood glue layers [154] and gelatin layers (biopolymer) [155].

### 4.4. Film Formation—Coalescence

In some applications, film formation involves coalescence (e.g., with latex particles). In case of latex dispersions, film formation occurs due to the interdiffusion of polymer chains rather then curing. Well-known applications are water-based paints [164,165,166,167] or pressure-sensitive adhesives [24,168].

The formation of dry, homogeneous films from colloidal dispersions such as latex in water can be described by a three-step process [22]. First, water evaporates and the particles concentrate and overcome their colloidal stability [169]. Next, the particles deform [170], trying to fill the void volume in the film. Lastly, interdiffusion of individual polymer chains overcomes the particles’ boundaries, forming a continuous and rigid film [171]. The deformation and interdiffusion of these polymers depens on the mobility of the latex particles, reflected by their glass transition temperature ($t_{g}$) [107]. The sequence of events can overlap in time. The processes can also influence each other. For instance, the water fraction will influence the mobility of the polymer chains [107,172], leading to a decrease in deformation and interdiffusion upon drying [85]. Studying these processes with NMR can give crucial information, leading to a better understanding of unwanted effects such as film cracking [173], trapped water [24] and irregular particle formation [174].

**Figure 10:**
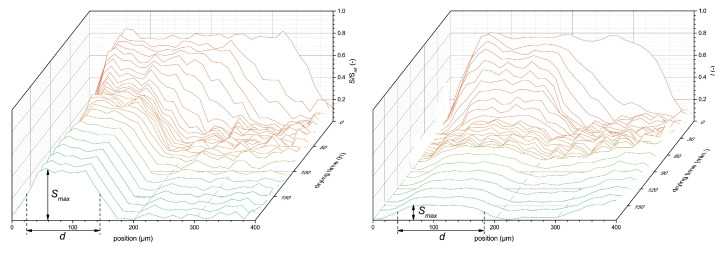
NMR signal intensity profiles during drying of soft- (**left**) and hard-type (**right**) latex. Indicated by $S_{max}$ and *d* are the final maximum signal intensities and thicknesses of the coatings. (Reprinted from *Progress in Organic Coatings*, Volume 123, Benjamin Voogt, Henk Huinink, Bart Erich, Jurgen Scheerder, Paul Venema and OlafAdan, Water mobility during drying of hard and soft type latex: Systematic GARField ${}^{1}$H NMR relaxometry studies, Pages 111–119, Copyrights 2018, with permission from Elsevier [107]).

To illustrate the use of high-resolution NMR for latex film formation, we use a study by B. Voogt et al. [107] as an example. In this study, drying of both soft ($t_{g}<$ room temperature) and hard ($t_{g}>$ room temperature) latices was studied inside a GARField NMR. Measurements were performed with an OW-sequence with $t_{e}=2\tau =40$ µs. Figure 10 shows profiles measured during the drying of both soft- (left) and hard-type (right) latices at an RH of 80%. As discussed in Section 4.2, a step-wise process is observed where first evaporative drying takes place. This will induce film shrinkage due to the evaporation of water that can be observed via a receding front. This will lead to higher concentration of latex particles. In both films, thickness (*d*) and maximum signal intensity ($S_{max}$) decreased over time. It was observed that the hard-type latex dried much faster. Hard-type latex thickness decreased until 20 min, while for the soft latex, this only happened at 40 min. The maximum intensity of the hard-type latex also lowered faster and ended lower at around 0.2, while for the soft latex a signal intensity of 0.6 was observed, both leveling off at 40 min. The difference in leveling off between $S_{max}$ and *d* for hard latex suggests a continuation of bulk water evaporation, while the latex particles are fixed in position and are not able to keep concentrating. This is understandable as particles below their $t_{g}$ will form brittle and porous networks [22] and are unable to form a continuous film.

In this study by B. Voogt et al., the protons in free water and polymer where studied simultaneously, using a multi-exponential decay analysis Section 2.2. A typical OW-decay is shown in Figure 2 right. For both latices, a clear multi-exponential decay was observed where the short relaxation time could be linked to the polymer and the long relaxation time to the free water phase. Both short relaxation times are fixed at 0.1 ms, showing that both latices have proton pools with mobilities that are not affected by drying. These are the protons embedded within the latex.

Using this multi-exponential decay, the different concentrations could be followed over time. The researchers found that a loss of free water ($\rho_{long}$) due to evaporation is independent of the lattice type. However, the latex fraction shows an increase for the soft latex type ($\rho_{short}$), indicating a further increase in concentration. This effect was not observed in the hard latex type. The increase in proton density is the consequence of coalescence of the particles. When film formation happens above the polymers t${}_{g}$, the polymer mobility is high enough that the polymer chains can interdiffuse and form a polymer film. This indicates that the t${}_{g}$ of latex has a huge influence on the film formation processes and the coalescence of the particles. This study illustrates that coalescence of latex particles can be made visual inside the NMR setups. This makes it possible to study the effect of multiple parameters on the coalescence kinetics and improve latex film applications.

**Figure 11:**
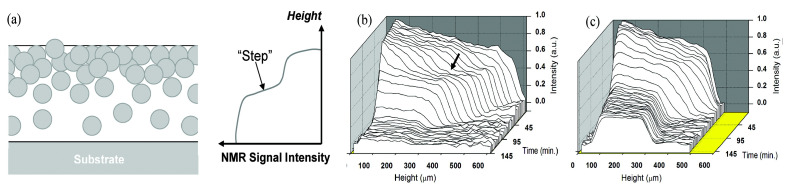
(**a**): schematic representation of latex packing near the surface accompanied by a representation of the corresponding NMR signal. NMR profiles measured over time for (**b**) acrylic copolymer latex (SM0) and (**c**) 25 wt% PDMS on the acrylic monomer(SM25) (Reprinted with permission from *Macromolecules* 2012, 45, 4, 1937–1945. Copyright 2012 American Chemical Society [175]).

Other studies have focused on the coalescence of particles at the liquid–air interface, leading to a ”skin-layer” [24,175]. In certain compositions, coalescence appears near the liquid–air interface during the drying stage. This is schematically represented in Figure 11 left. This skin-layer will block the evaporation, leading to trapped water within the film. R. Rodriguez et al. [175] studied the film formation for different compositions: acrylic copolymer (SM0), a hybrid latex containing 25% PDMS (SM25), and a blend of the acrylic (SM0) with 11 wt% PDMS emulsion. Drying measurements on two of these compositions are shown in Figure 11, namely, SM0 (b) and SM25 (c). The SM0 polymer has a much lower mobiltiy than the SM25. Therefore, the signal intensity for SM0 is almost zero after drying, while for the SM25 there is still signal. By setting the echo time to 180 µs, the researchers made sure that almost no signal was attributed to the SM0 polymer. The signal could be attributed to free water. In both drying experiments, the signal intensity increases in the depth of the film. This increase was also observed in other studies [24] and was attributed to a gradient in free water. In the neat acrylic dilution (SM0), a deviation from this linear behavior is observed where a layer with lower signal intensity appears at the top. This indicates a step in the free water concentration and a denser packing of latex particles at the surface (Figure 11a). This layer increased in thickness over time. This drastic step in particle density was not observed in the SM25, see Figure 11c. Here, a more uniform profile is observed that reached its final form at 55 min.

Other studies used similar NMR measurements to study the effect of glass transition temperature [176], surfactant [168], tackifying resins (TR) [24], salts [177], different amounts of carboxylic acid functional groups [105] and the Peclet number [151] on the film formation process. GARField NMR seems to be the best technique for these processes, as it has a high-enough FOV to cope with the latex films and can give the best resolution. However, measurements with a MOUSE were also performed on latex films [120]. This, however, led to lower resolutions (30 µm) and was hampered by long measurement times.

### 4.5. Diffusion/Penetration

Liquid uptake and diffusion in thin layers is important for coatings, the printing industry or sustainability of materials. Water and solvents can weaken interactions between neighboring polymers and ultimately lead to failure of the thin layer by softening or cracking. Both the amount of liquid and the speed of penetration are crucial parameters in characterizing the stability of thin layer. In this section, it will be shown how NMR profiling has been used to measure liquid penetration over time. A crucial parameter for solvent penetration is the diffusion coefficient. This parameter is, however, difficult to measure because it depends on morphology such as crystallinity [178] or temperature.

To illustrate the capability of high-resolution NMR depth profiling in studying penetration and diffusion experiments, a study by N. J. W. Reuvers et al. [179] is used. The water uptake in thin nylon-6 films was measured with a GARField NMR.

**Figure 12:**
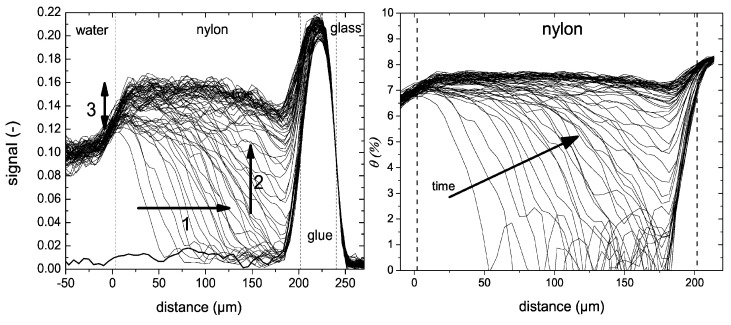
**Left**: Liquid profiles measured during the uptake of water into 200 µm thick nylon membranes. **Right**: same profiles as in the middle graph but after replacing the signal intensity with the moisture content. (Reprinted with permission from *Macromolecules* 2012, 45, 4, 1937–1945. Copyright 2012 American Chemical Society. [179]).

The water uptake was measured using an OW-sequence. A water uptake experiment in a 200 µm thick nylon-6 is shown in Figure 12 left. When going from right to left, the glass plate, glue layer, nylon and water can be observed which can also be seen in the schematic picture of the setup. While the glass plate cannot be imaged by the NMR, a clear difference between the silicon glue, nylon film and water is observed. The observed differences in signal intensities can be linked to varying $T_{1}$ and $T_{2}$-times. Shown with a bold line is the signal intensity measured before the experiment. The researchers distinguished three different processes. (1) A liquid fronts develops, traveling towards the bottom of the layer (t < 6 h), (2) water distributes equally over the film (6 h < t < 10 h) and (3) a slower processes occurs where a small signal increase is observed near the glass–polymer interface. Using these profiles, the researchers determined the actual diffusion coefficient within the layer. To be able to do this, the researchers needed to convert the NMR signal intensity to moisture content (θ). They could link the NMR signal to a moisture profile using gravimeter calibration [179]. The resulting relationship was nonlinear and was attributed to plasticization and a change in relaxation of free water. The NMR signal profiles in Figure 12 can be viewed as a supper position of a liquid front and plasticization front. Using the relation between moisture content and NMR signal, the original NMR profiles (Figure 12 left) are converted to moisture content profiles (Figure 12 right). The small signal increase observed before (3) could be attributed to polymers that become more mobile and contribute to the signal.

**Figure 13:**
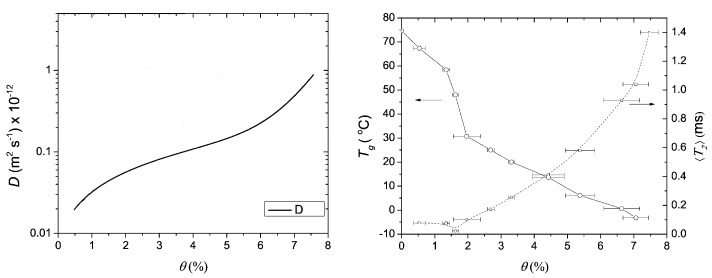
**Left**: the diffusion coefficient (*D*) in function of moisture content. **Right**: The glass transition temperature ($t_{g}$) and average relaxation time ($T_{2}$) in function of moisture content. (Reprinted with permission from *Macromolecules* 2008, 41, 22, 8537–8546. Copyright 2012 American Chemical Society [175]).

The moisture profiles were used to extract the diffusion coefficient of water within the nylon film. The calculated diffusion coefficients are shown in Figure 13. The diffusion coefficient increases with increasing moisture content.

To study the signal change due to plasticizing, the excess water is replaced by D${}_{2}$O. D${}_{2}$O has the same characteristics as water but will not contribute to the NMR signal. It was observed that half the NMR signal in the profiles was linked to hydrogen atoms of water and half of the signal to mobilized polymer. Additionally, the glass transition temperature and $T_{2}$-relaxation could be linked with moisture content (Figure 13 right). In a follow-up study, it was found that the plasticization lags behind the water migration in these nylon-6 thin films [180].

Similar penetration studies have been performed on dental resins. Using STRAFI, G. Hunter et al. [158] studied the uptake of water and water/ethanol mixtures into a commercial dental resin. Measurements with different ethanol concentrations revealed that the diffusion coefficient increased with ethanol content. Additionally, transport in ceramic substrates [181], multilayer coatings [141,182], glassy pellets of the starch polymer amylose [124], cement pastes [136] glue lines [154], vulcanized rubber [183] and nylon-6 [140,141] have been studied. In addition to the penetration of water, the transport of different ions such as Mn${}^{2+}$ and Cu${}^{2+}$ [184], different salt solutions [185] and the ingress of vapours [186,187] have also been studied with the NMR setups. Finally, the influence of stress [188] on the penetration behavior was studied by V. Baukh et al.

### 4.6. Thin Films on Site: Cultural Heritage

In some applications, measurements of thin layers need to be performed outside the lab because it is impossible to collect small samples or to recreate a similar structure. As the STRAFI and GARField setups are located within the lab and require samples of a specific shape and size, measurements on site are performed by NMR-MOUSE. The portability of the NMR-MOUSE makes it possible to measure samples with infinite lateral dimensions where limits are mostly bound because of time constraints. One big category of such applications studied with the NMR-MOUSE is cultural heritage [189]: for example, in case of paintings on wood. A significant issue with cultural heritage is structural damage during conservation. Using the NMR-MOUSE, the internal structure of paint layers can be studied. These studies can help in characterizing different sources of damage and identifying the best conservation environment.

Damage of cultural heritage is mainly caused by moisture uptake which, for example, in wooden painting or walls, can cause degradation over time. Measuring the moisture distribution can provide information on the conditions in which the painting should be stored and can help in preserving the cultural heritage [8]. In this section, a closer look at some analysis methods will be given, demonstrating the use of the NMR-MOUSE on cultural heritage.

**Figure 14:**
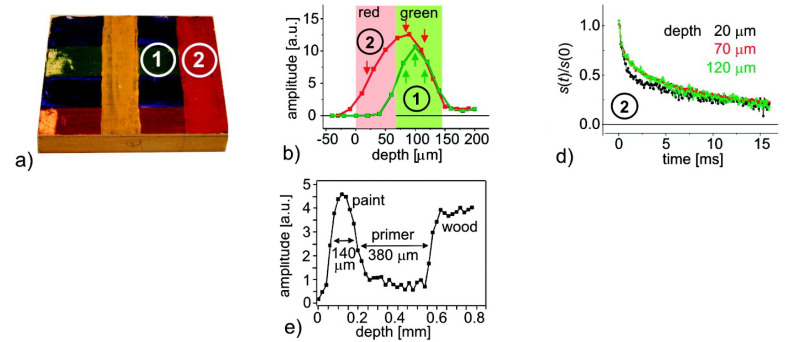
(**a**): Easel painting model with a wooden background covered by a primer and one (1) or two (2) paint layers. (**b**): measured depth profiles for the position marked in Figure 14a. A clear difference in thickness is observed between both layers where one layer is thinner than the two layers. (**d**) Signal decay measurements at position 2 for different depths. (**e**) Complete depth profile through the painting indicating the different layers. (Reprinted with permission from *Acc. Chem. Res.* 2010, 43, 6, 761–770. Copyright 2010 American Chemical Society [7]).

B. Blumich et al. [7] demonstrated the use of the NMR-MOUSE for studying the layered structure of paintings. They started with a wood panel covered by a primer and one or multiple layers of paint. They tried to mimic the structure found in old paintings. Figure 14 shows a picture of the structure where two positions are marked where either one (1) or two (2) layers of paint are used. Depicted in Figure 14b are the depth profiles measured by an NMR-MOUSE of the two sides shown in Figure 14a. Both paint layers had good signal and the thickness of the layers is also reflected in the width of the NMR signals. Imaging different layers was performed both with total amplitude measurement and characterizing the signal decays (CPMG). In Figure 14e, the total NMR signal is measured along the thickness of the painting revealing a layered structure where paint, primer and wood are observed. Using this method, the thickness of the different layers could be monitored at different points, revealing damage at certain positions. The CPMG could give information about changes within the layers over time.

In the same paper, the authors also demonstrated the ability to characterize damaged old paper structures by measuring the hydrogen atoms in the cellulose fibres, time-dependent water uptake and drying in stone. Even old master violins such as a Stradivari were investigated in this way. The study revealed that the master violins’ wood density increased with age, which determined the quality of the instrument. The possibilities of this measurement technique was also used for the ancient Roman fresco and the bricks in the walls of the cryptoporticus at Colle Oppio in Rome [190], the degradation of historical paper [76] and the conservation treatments on paintings [191,192,193].

### 4.7. Conclusions

Three different high-resolution NMR setups able to study thin layers between 10 and 1000 µm were reviewed, namely, STRAFI, GARField and MOUSE. These NMR techniques are all able to give spatial and time-resolved information about thin layers.

The setups used two different acquisition methods: slice-selective pulse and Fourier-based measurements. In the slice-selective pulse measurements performed by MOUSE and in conventional STRAFI, the signal is recorded in a step-wise manner, whereby the pulse excites the region of interest. In this case, the resolution is determined by the pulse length given by $\Delta r\approx 1/t_{p}\gamma G$. High resolution can be achieved at the expense of long measurement times. The advantage of the step-wise acquisition is that sample size is only limited by the aperture used. Unfortunately, the resolution is limited by the large curvatures found in the main magnetic field $\vec{B_{0}}$ of both STRAFI and MOUSE and should be determined experimentally. In GARField and in some cases in STRAFI, the acquisition is achieved by a Fourier analysis. The sample is excited with one pulse (limiting the total measurement area) where the resolution is determined by the acquisition time, given by $\Delta r\approx 1/t_{a}\gamma G$. As the sample is measured using one pulse, measurements are much faster. In GARField, the problem with the inhomogenous field is solved by specially designed poles leading to the best space and time resolution found in all the setups. Moreover, Fourier STRAFI exists, which can measure much faster then conventional STRAFI. However, due to the arrangement of the magnetic field, a solenoid-shaped coil is needed that compared to the surface coil in GARField will always have lower sensitivity. A drawback of this special design is that GARField lacks the ability to measure samples larger then 400 µm. To measure larger samples, STRAFI would be more appropriate. Finally, the NMR-MOUSE has proven to be valuable in cultural heritage because it is the only mobile device able to measure depth profile on-site in a non-invasive manner.

A variety of applications such as drying, film formation, penetration and measurements on cultural heritage have been reviewed illustrating the potential of these NMR-profiling tools. By measuring hydrogen profiles over time, the setups were able to follow drying, curing and penetration fronts. Using these profiles, the diffusion coefficient and front kinetics could be determined for a wide variety of parameters. Detailed structural information about thin layers could be gathered by measuring $T_{2},T_{1}$ relaxation times and signal intensity. The information could be used to follow film formation, film degradation, penetration and diffusion experiments.

The current measurement techniques lack the ability to measure below 1 min. Future improvements towards higher time resolutions is, for example, useful in the printing industry where the penetration occurs at timescales of 100 ms. Great potential was shown by R. J. K. Nicasy et al. [194] that demonstrated the use of high-speed NMR to profile liquid uptake in non-transparent porous media with time resolutions of 10 ms. This was only shown for liquid penetration but could be extended towards the characterization of chemical structures. In addition to measuring speed, improvement towards the amount of chemical information obtained with the setup can be achieved by combining the above-mentioned techniques with NMR spectroscopy. Currently, no such studies are available, but they could be very valuable to determine chemical components and reactions in much more detail. Lastly, by combining the NMR techniques with other measurement setups, a better understanding of the information can be achieved. This has already been shown to be valuable with confocal Raman microscopy [89] and electrochemical impedance spectroscopy [195], but could be extended towards other techniques.

## Figures and Tables

**Table 1:** The table summarizes the most important characteristics of the different setups.

	|$\vec{B_{0}}$|	|$\vec{G}$[T/m]|	Measure Time	Resolution	Portable	Sample Size
STRAFI	2.3–7	30–60	>3 min	24–60 µm	no	limitless
GARField	0.7–1.4	17–44	1–10 min	5–15 µm	no	50–400 µm
MOUSE	0.025–0.7	11.5	60 min	50–1000 µm	yes	100–4000 µm

## Data Availability

Not applicable.
